# Comparison of Short-term and Long-term Outcomes after Different Reconstructions between Totally Laparoscopic Distal Gastrectomy and Laparoscopic-assisted Distal Gastrectomy for Gastric Cancer: a Retrospective Analysis at a High-volume Center

**DOI:** 10.7150/jca.97786

**Published:** 2024-07-16

**Authors:** Qingya Li, Mengpei Yan, Fengyuan Li, Zheng Li, Linjun Wang, Diancai Zhang, Hao Xu, Zekuan Xu, Sen Wang

**Affiliations:** 1 Department of General Surgery, The First Affiliated Hospital of Nanjing Medical University, Nanjing, 210029, China.; 2 Gastric Cancer Center, The First Affiliated Hospital of Nanjing Medical University, Nanjing, 210029, China.; 3 The Institute of Gastric Cancer, Nanjing Medical University, Nanjing, 210029, China.; 4 Collaborative Innovation Center for Cancer Personalized Medicine, Nanjing Medical University, Nanjing, 210029, China.

**Keywords:** Gastric cancer, Laparoscopic-assisted distal gastrectomy, Totally laparoscopic distal gastrectomy, Complications, Postoperative hospital stay.

## Abstract

**Background:** The short-term and long-term outcomes of laparoscopic-assisted distal gastrectomy (LADG) and totally laparoscopic distal gastrectomy (TLDG) have been subject to controversy with various reconstruction techniques of Billroth-I, Billroth-II, Roux-en-Y, and Uncut. This study aims to compare the short-term and long-term outcomes of LADG and TLDG as well as the outcomes of different anastomoses.

**Methods:** This study enrolled patients with gastric cancer at the First Affiliated Hospital of Nanjing Medical University (NMUH) between 2017 and 2021. Postoperative complications were classified according to the Clavien-Dindo grade. Exclusion criteria included metachronous and synchronous malignancy and palliative surgery. The Kaplan-Meier analysis was applied to assess 5-year prognosis between two groups.

**Results**: This study included 1221 cases with an overall complication rate of 17.37% for LADG, which was significantly higher than TLDG's 10.72%. The incidence of anastomosis-related complications was 4.79% for LADG and 1.13% lower for TLDG. LADG and TLDG did not show significant difference for Grade III-V complications and resected lymph nodes. The postoperative stay was shorter for TLDG than LADG, and R-Y had a longer postoperative stay than B-II and Uncut after combining LADG and TLDG. The operation time was shorter in TLDG cases than that in LADG cases. The 5-year OS of the TLDG group was not significantly better than that of the LADG group.

**Conclusion**: TLDG is superior in overall complication rate, anastomosis-related complication rate, postoperative stay and operation time to LADG. No difference of OS was observed between LADG and TLDG. Four anastomoses had no convincing evidence of being superior in complications rates, post-op stay, and harvested lymph nodes to each other.

## Introduction

Gastric cancer is now the 5^th^ most prevalent malignancy worldwide and caused the 4^th^ highest cancer-related mortality worldwide according to the latest cancer statistics^1^. Presently, the comprehensive treatment approach for gastric cancer encompasses surgical interventions, chemotherapy, radiotherapy, and other modalities, with surgery remaining the sole radical treatment option^2^-^5^. The advent of laparoscopic surgery has notably advanced the landscape of gastric cancer surgical management, with laparoscopic distal gastrectomy emerging as a viable and safe alternative. KLASS-01 RCT have confirmed that efficacy and safety of laparoscopic surgery in early gastric cancer with similar oncological safety compared to open surgery^6^. Additionally, studies such as KLASS-02 and CLASS-01 indicated the feasibility of laparoscopic distal gastrectomy in locally advanced gastric cancer with no inferior outcomes than open distal gastrectomy^7^, ^8^. Laparoscopic-assisted distal gastrectomy (LADG) is first been applied in distal gastrectomy compared to totally laparoscopic distal gastrectomy (TLDG) since Kitano et al reported the first case of laparoscopic distal gastrectomy^9^. The gastrojejunnal anastomosis in LADG is performed extracorporeally with a wider incision. The gastrojejunnal anastomosis in TLDG is performed intracorporeally with a more demanding laparoscopic skill manipulating a linear or a circular stapler and a better surgical view. However, even though both types are commonly performed, there is still some controversial on outcomes and survival rates between LADG and TLDG. Some studies have shown better outcomes after TLDG, while others suggest no significant difference between each other 10-14. Consequently, a definitive preference between LADG and TLDG remains elusive.

Reconstructions after laparoscopic radical distal gastrectomy predominantly encompass Billroth I, Billroth II, Roux-en-Y and Uncut Roux-en-Y anastomosis, performed either in a totally laparoscopic or laparoscopic-assisted manner. Debates between each other regarding complications of these 4 reconstructions have also been lasting with no consensus. The selection of anastomosis basically depends on the surgeons' preference. Most studies have reported that there are no significant differences regarding complications and quality of life when comparing laparoscopic Billroth I, Billroth II and Roux-en-Y with only a few studies showing Roux-en-Y demonstrated superior outcomes to Billroth I and Billroth II^15^-^23^. According to Korean Gastric Cancer Guideline, Gastroduodenostomy and gastrojejunostomy (Roux-en-Y and loop) are recommended after DG in middle and lower gastric cancer. There are no differences in terms of survival, function, and nutrition between the different types of reconstruction^24^. However, there are not enough reports comparing laparoscopic uncut Roux-en-Y and other methods since it is a comparatively new technique with fewer cases than the conventional methods. Although previous literature has compared patients undergoing Roux-en-Y reconstruction following total laparoscopic distal gastrectomy (TLDG) and laparoscopy-assisted distal gastrectomy (LADG) in terms of surgical time, short-term postoperative complications, and other related outcomes, except for less blood loss in the TLDG group compared to the LADG group, there were no statistically significant differences between the two groups in surgical time, lymph node dissection, time to first flatus, time to oral intake, postoperative hospital stay, and other aspects^25^. To update, no reports have compared all four methods with a LADG or TLDG way.

In this study, our objective is to retrospectively compare the short-term and long-term outcomes including total complications, grade III-V complications, anastomosis-related complications, postoperative hospital stay, and resected lymph nodes and overall survival between LADG and TLDG and among all 4 different anastomosis retrospectively. This endeavor aims to contribute additional evidence to aid in the selection of appropriate anastomosis methods following laparoscopic distal gastrectomy.

## Methods

### Patients

Patients diagnosed with gastric cancer and treated at the Gastrointestinal Surgery Center, the First Affiliated Hospital of Nanjing Medical University (NMUH), between 2017-2021 were enrolled in this study. Pathologic conformation of stomach adenocarcinoma in all patients was based on the 8^th^ AJCC pathology TNM staging system. The study encompassed patients who underwent laparoscopic distal gastrectomy with various anastomosis techniques, including Billroth I, Billroth II, Roux-en-Y, and Uncut Roux-en-Y, utilizing either totally laparoscopic or laparoscopic-assisted approaches. To be noted, B-II includes all cases with or without Braun anastomosis. The exclusion criteria include metachronous and synchronous malignancy and combined resection of other main organs. Palliative surgeries are removed from the database. The conversion to open surgery was not included in the study.

### LADG and TLDG

We defined intracorporeal gastrojejunal or gastroduodenal anastomosis with an incision less than 6 cm as TLDG and extracorporeal gastrojejunal or gastroduodenal anastomosis as LADG.

### Data collection

The complication categories include: duodenal stump leakage, anastomotic leakage, anastomotic bleeding, intraabdominal bleeding, intestinal obstruction, abdominal infection, seroperitoneum, wound infection, lymphatic fistula, anemia, mobility disorder, cardiac complications, pulmonary complications, urinary and renal complications, hepatobiliary complications, other gastrointestinal complications and thrombosis. Residents and statisticians are constantly recording the data. Postoperative complications that occurred within 30 days after surgery were assessed according to Clavien-Dindo classification.

### Statistical analysis

We noticed that in some methods the cases number was quite small, which can lead to analytical bias. It's noted that in some methods, the number of cases was relatively small, potentially introducing analytical bias. So when compared various anastomoses in LADG, we did not take B-I into analysis due to lack of cases. We did not calculate *p* value of comparison with B-I in after combining LADG with TLDG for the involvement of LADG B-I. The statistics software SPSS 25 was adopted for the analysis. Mann-Whitney and t-test analysis are used throughout the study. Continuous variables are described as mean±SD or median(Q1,Q3), and categorized variables are summarized by frequency (n) and proportion (%). Chi-square test was used for rate or proportion comparison. Kaplan-Meier analysis were used to assess 5-year overall survival rates between groups. Statistical analyses were performed using SPSS software, ver. 25.0. P<0.05 indicates statistical significance. The univariate and multivariate analysis of variance was conducted by R. Written consent was obtained from all patients in NMUH and it was approved by the ethics committee of the First Affiliated Hospital of Nanjing Medical University.

## Results

### Overview

**Figure 1:**
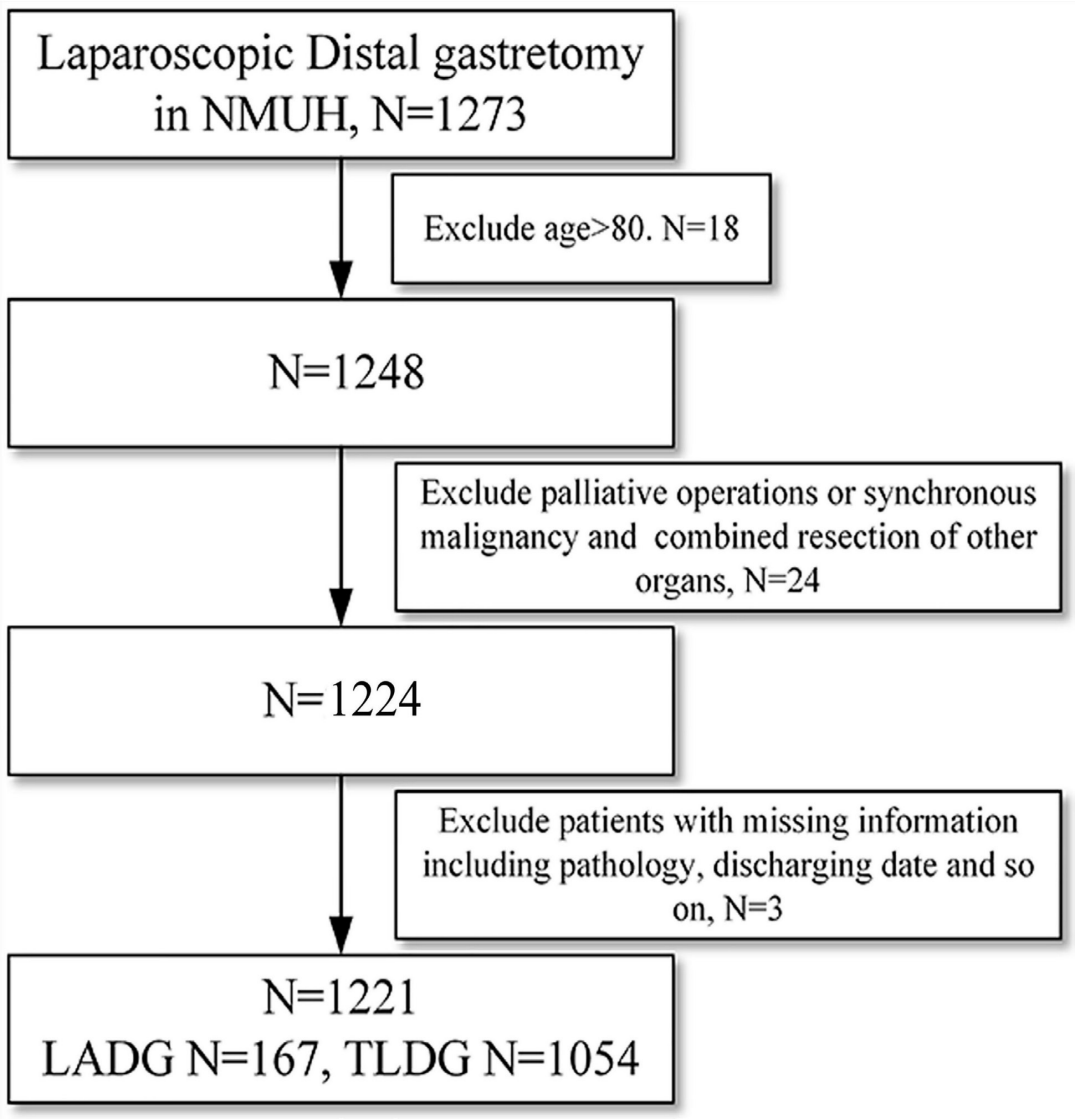
Flowchart of patients enrollment of the study.

After screening patients on the basis of inclusion and exclusion criteria (Figure 1), there were 1221 cases including 786 male and 435 female from NMUH enrolled in this study (Table 1). The age ranges from 26 to 80 in NMUH. There were 1, 49, 102 and 15 patients who received LADG B-I, B-II, RY and Uncut respectively. There were 11, 704, 101 and 238 patients receiving B-I, B-II, RY and Uncut anastomosis with TLDG respectively.

### LADG has a significantly higher overall complication rate than TLDG

The number of cases with complications (grade I to grade V) of all operations are listed in Table 2A. No mortality occurred in all cases of our study. However, due to the small number of cases involving LADG B-I (less than 10), it was not included in the analysis. Initially, it is noteworthy that the overall complication rate for all laparoscopic distal gastrectomy cases from 2017 to 2021 was 11.63%, suggesting the safety and feasibility of laparoscopic distal gastrectomy at our center. Subsequently, we conducted comparisons between different types of anastomoses within the LADG and TLDG subgroups, respectively. No significant difference was found among B-I, B-II, RY and Uncut in the LADG subgroup and in the TLDG subgroup Table S1. After combining the LADG and the TLDG cases, it indicated that there was no significant difference in overall complications among various anastomoses Table S1. Then we compared the LADG subgroup with the TLDG subgroup to examine whether LADG or TLDG could influence the outcomes. We found that overall complication rates did not significantly differ between LADG with TLDG in B-I, B-II, RY and Uncut anastomosis respectively. However, when we compared all cases of LADG with TLDG, TLDG showed a significantly lower complication rate with 10.72% than that of LADG with 17.37% (Table 2B).

We then listed all complication types of LADG and TLDG to determine which complications led to the difference Table S2. Interestingly, we observed higher rates of anastomotic leakage and bleeding in LADG compared to TLDG. These indicated the potential advantage of TLDG over LADG. Moreover, we noticed the rate of LADG wound infection was also higher than that of TLDG. As widely understood, a wider incision of LADG could cause a higher risk of wound infection and this proved again the importance and superiority of less invasiveness in gastrectomy.

We then performed univariate and multivariate analysis of variance to detect whether different methods of laparoscopy and different anastomosis could affect the overall complication rates or not Table S3. Both univariate and multivariate analysis indicated that TLDG and LADG had significant differences in the overall complication rate, which was consistent with previous results. We also did a univariate and multivariate analysis based on T stage and N stages and the results confirmed that T and N stages did not affect the overall complication rate, indicating that more advanced GC is not positively associated with higher overall complication rates. Generally, these results suggested a better outcome of TLDG than LADG in terms of decreasing overall complication rate.

### The rate of anastomosis-related complications is higher in LADG than in TLDG

Anastomotic leakage, duodenal stump leakage and anastomotic hemorrhage are the most serious complications of anastomotic fistula, and we treated these three types as anastomosis-related complications. The information about cases with anastomosis-related complications is listed in Table 3A. We did the same analysis as the previous complication analysis. No positive results emerged in anastomosis among B-I, B-II, RY and Uncut Table S1. When we compared TLDG cases with LADG cases, we again found that the complication rates did not differ between LADG with TLDG in B-I, B-II, RY and Uncut anastomosis respectively, but we found the anastomosis-related complication rate in all LADG with 4.79% is significantly higher than that of all TLDG cases with 1.13% (Table 3B). Although manipulating staplers intracorporeally is more difficult than extracorporeally, TLDG proved not only equal outcome in anastomosis but even better than LADG, as long as with adequate experience. This might suggest TLDG may be a better anastomotic option for reconstruction in laparoscopic gastrectomy.

The univariate and multivariate analysis of variance indicated TLDG and LADG had a dramatic difference in the anastomosis-related complication, which was also consistent with previous results Table S4. Neither T or N stage would significantly affect the outcomes, showing that these stages were not quite associated with the anastomosis-related complications. These results confirmed again that TLDG might have a potential advantage for anastomosis than LADG.

### TLDG is not superior to LADG in grade III-V complications

Then, we selected cases only with complications rated as grade III-V according to the Clavien-Dindo grade system. We consider complications with grade III-V to have a better correlation with the quality of anastomosis and surgery. The basic grade III-V complications information is listed in Table 4A. Again, we did the same analysis as the previous ones. In LADG subgroup and TLDG subgroup, no significant difference was observed among various anastomosis Table S1. This is consistent with our previous conclusion. When comparing all anastomosis with each other after combining LADG and TLDG, negative outcomes showed that different anastomosis would not affect grade III-V complications rate Table S1. Then we compared laparoscopic-assisted anastomosis with totally laparoscopic anastomosis cases respectively. We found that LADG B-II, RY and Uncut had no significant difference with TLDG B-II, RY and Uncut. For all cases, overall LADG cases with 2.99% grade III-V complication rate did not differ from overall TLDG cases with 2.94% grade III-V complication rate (Table 4B), indicating no anastomosis would influence the grade III-V complications.

The univariate and multivariate analysis of variance demonstrated that, like previous analysis, T and N stage did not change the grade III-V complication based on the multivariate analysis of variance Table S5. LADG or TLDG also made no difference in the grade III-V complication rates, according to the analysis. This provided more solid evidence for our results.

### The length of postoperative stay after TLDG is shorter than that of LADG

We also calculated length of postoperative hospital time to indicate the quality of recovery of different methods and anastomosis. Since the distribution of postoperative stay is not a normal disruption, we used Median (lower quartile, upper quartile) to indicate the post-op hospital stay. The detailed data of postoperative time is listed in Table 5A. When comparing different anastomosis in the LADG subgroup, we found that the length of postoperative hospital stay of RY was significantly longer than that of B-II but has no significant difference with Uncut Table S6-7). In the TLDG subgroup, we found that B-I had longer postoperative stay than B-II and Uncut (Table S6, S8). When we combined TLDG and LADG, it showed that RY had a significant longer length of postoperative stay than B-II and Uncut respectively (Table S6, S9). When comparing the LADG subgroup with the TLDG subgroup, it is surprising to find that all TLDG methods demonstrated better postoperative hospital stay than LADG methods, including B-II, RY and Uncut separately and all LADG and TLDG cases (Table 5B. These results suggest that TLDG might demonstrate a better effect in recovery than LADG, which was consistent with previous analysis.

### The operation time of enrolled cases in TLDG was significantly shorter than that in the LADG group

Regarding the operation time, we found that the operation time of TLDG RY and TLDG Uncut cases was significantly shorter than those of LADG cases, respectively (RY: 178.32±47.79 vs 238.6±59.55, p<0.0001; Uncut: 173.79±35.99 vs 199.07±43.72, p=0.043). The operation time of all TLDG cases was also significantly shorter than all LADG cases (174.13±37.78 vs 219.71±60.34, p<0.0001) (Table 6A-6B). We did the subgroup analysis too and found that operation time of LADG BII, RY and Uncut were also significantly different and LADG RY demonstrated longer time than others while no difference was found between LADG BII and Uncut Table S10-11). For TLDG cases, the subgroup analysis did not reveal significant difference on operation time among TLDG BII, TLDG RY and TLDG Uncut (Table S10.

### The long-term prognosis of the TLDG and LADG was not significantly different

**Figure 2:**
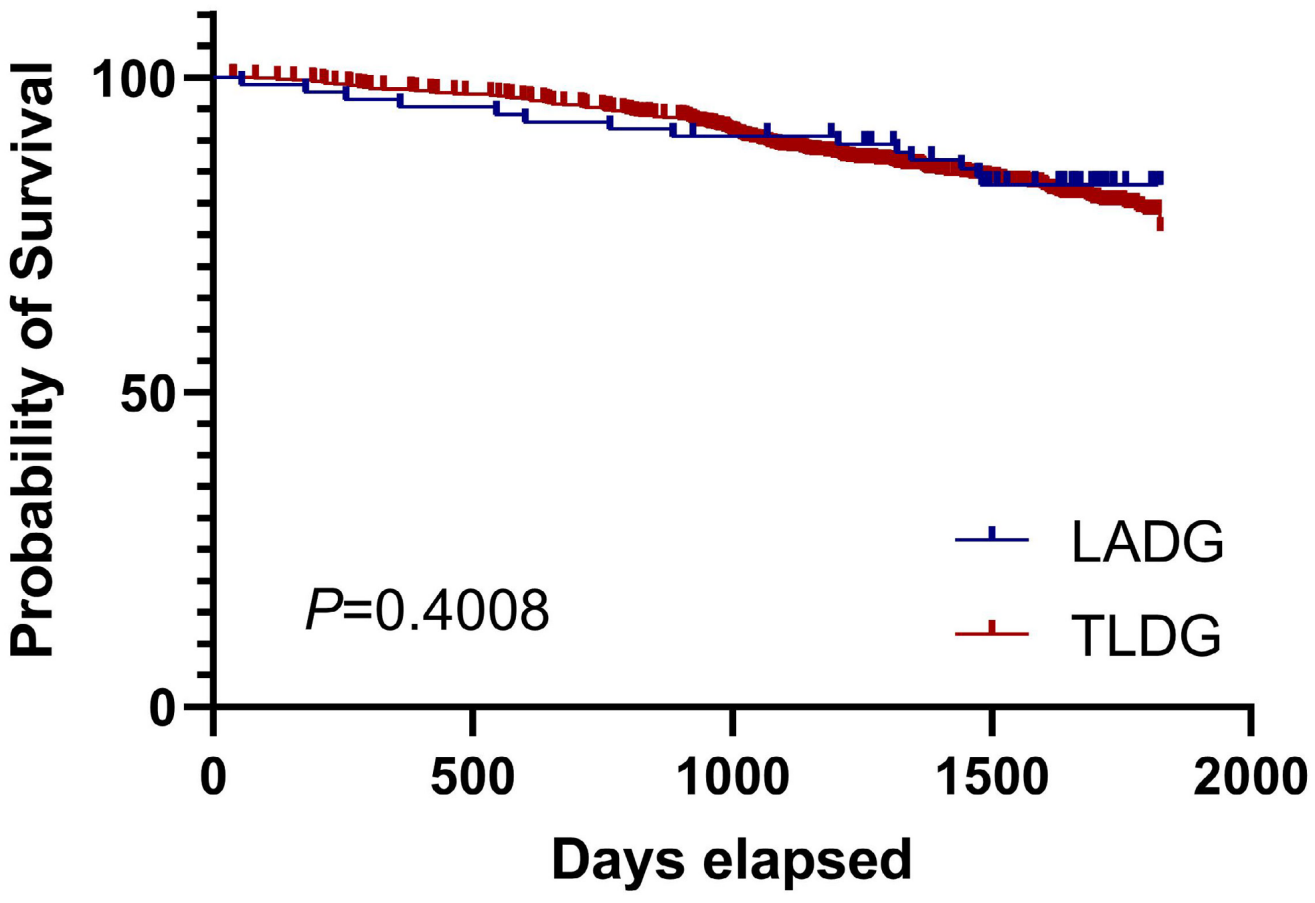
Overall survival of all patients in LADG and TLDG groups.

**Figure 3:**
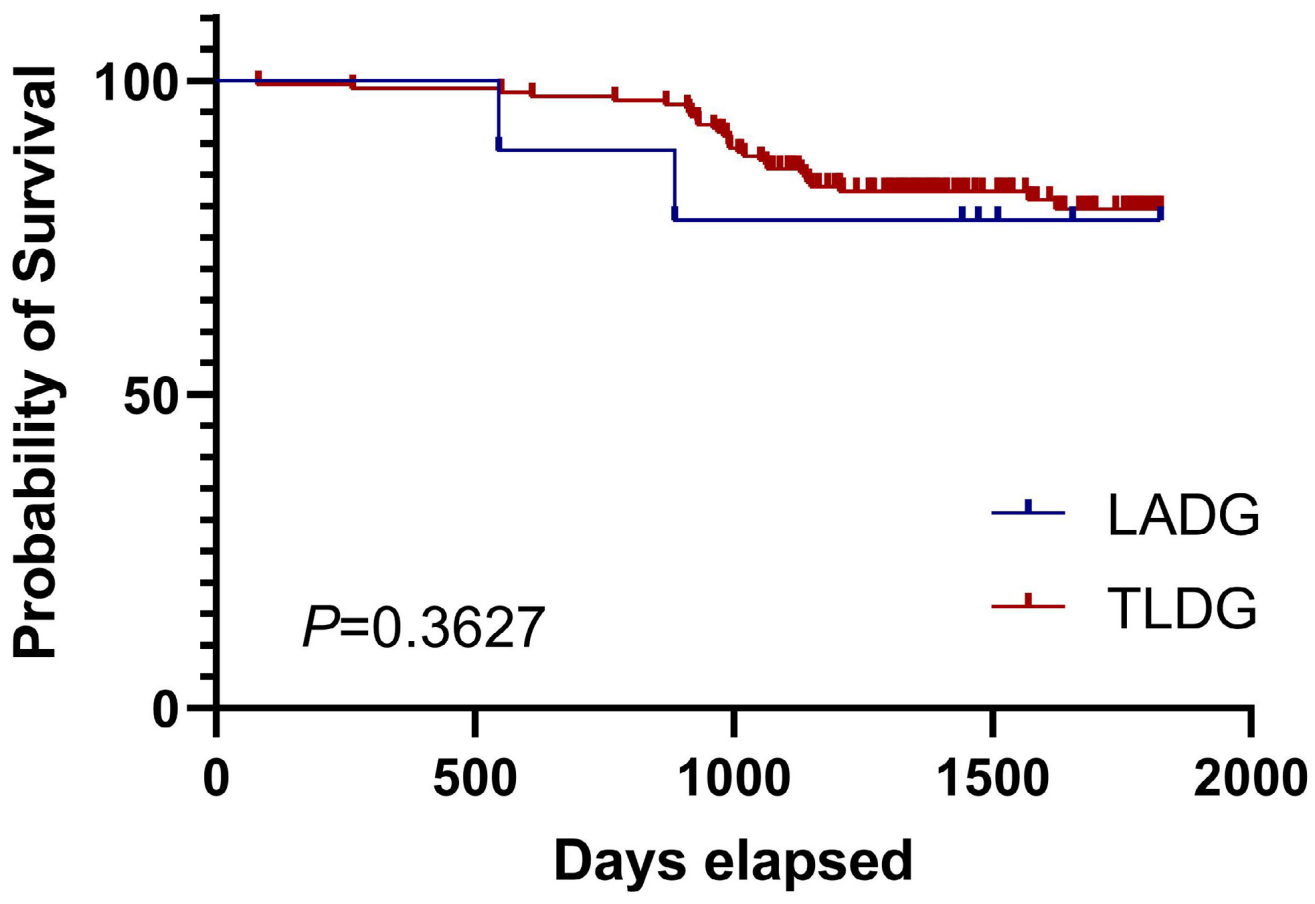
Overall survival of stage Ⅱ patients in LADG and TLDG groups.

**Figure 4:**
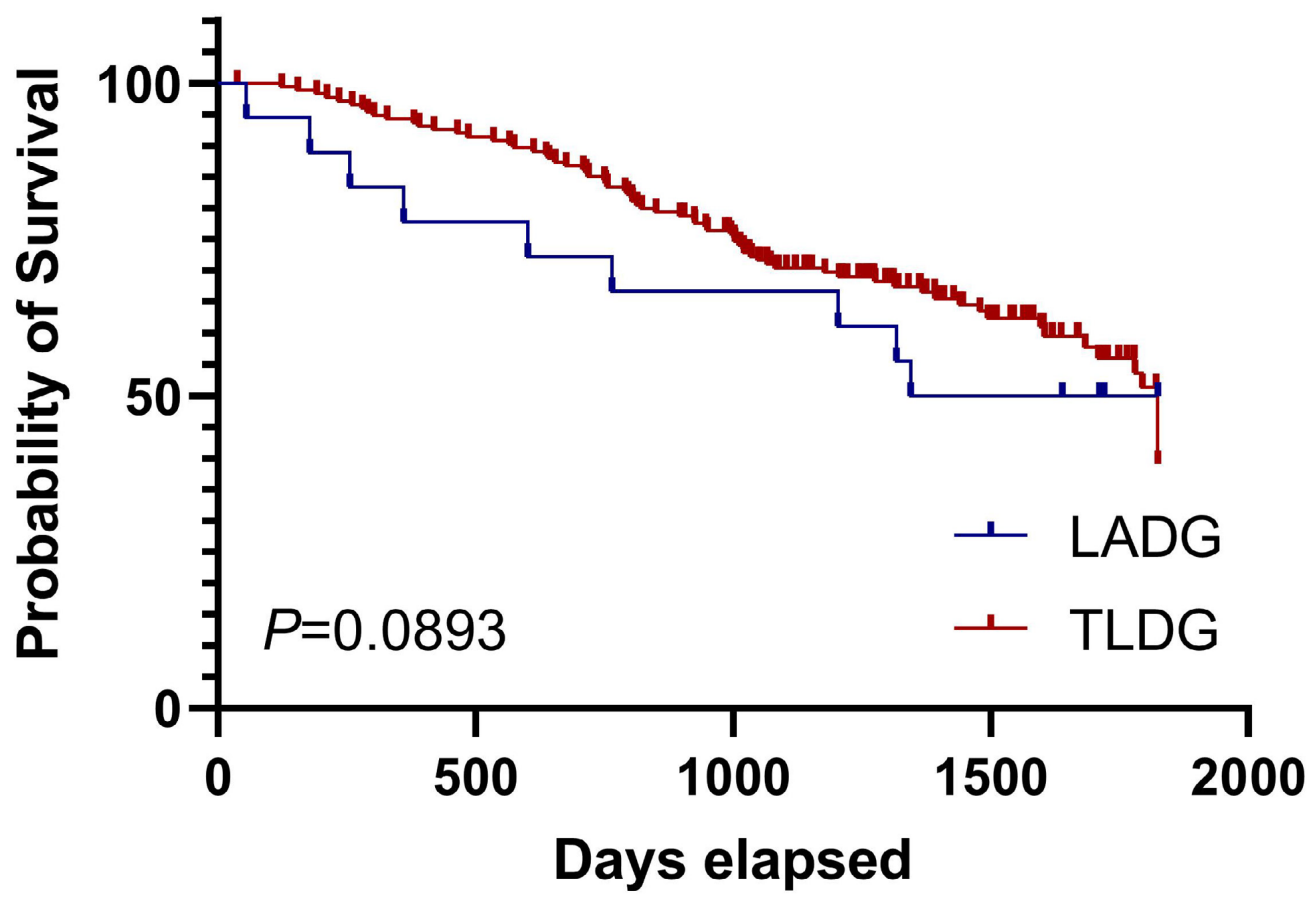
Overall survival of stage Ⅲ patients in LADG and TLDG groups.

We finally analyzed the long-term overall survival (OS) of LADG and TLDG patients. The 5-year prognosis of both groups was observed. We noticed that the OS of the TLDG group was not statistically different from that of the LADG group and the median survival time was not reached (Figure 2). Then we conducted OS analyses based on different stages. Due to the small number of stage I patients, the results were not demonstrated. Survival curves for stage II patients showed that TLDG seemed to have slightly better OS than LADG (Figure 3), without achieving the median survival time. But the difference between the two does not have statistical significance. For stage III patients, survival curves showed that although the OS of TLDG seemed slightly better than that of LADG, there was also no significant statistical difference between TLDG and LADG patients (Figure 4). Overall, the 5-year OS of the TLDG group was not significantly different than that of the LADG group.

## Discussion

Both LADG and TLDG are laparoscopic digestive tract reconstructions performed by experienced surgeons worldwide. In LADG, gastrointestinal or gastroduodenal anastomosis is conducted extracorporeally, necessitating a wider incision on the upper abdomen to pull out the remnant stomach. While this approach may pose challenges in patients with obesity or limited visibility, it mirrors open anastomosis techniques and can be executed proficiently by surgeons with extensive experience in open surgery.

Conversely, in TLDG, gastrointestinal or gastroduodenal anastomosis is performed intracorporeally, offering a clearer surgical view and requiring smaller abdominal incisions compared to LADG. However, the maneuvering of linear or circular staplers with precise margins may present increased complexity, potentially leading to higher rates of anastomosis-related complications. The controversy between LADG and TLDG persists due to conflicting reports and varying support from surgeons. Toshihiko *et al.* reported that TLDG has no significant difference on complications than LADG but anastomosis-related complications in TLDG may lower than LADG and postoperative stay is shorter in TLDG^14^. Won Ho Han *et al.* suggested that even though TLDG demonstrated better outcomes in blood loss and postoperative pain, their complications rate and postoperative time has no significant difference^11^. Ke Chen *et al.* also suggested that TLDG is safe and feasible, but no clinical advantages of TLDG was demonstrated in their study over LADG^26^. Woo J *et al.* suggested that although the proximal resection margin was significantly longer and the length of wound was shorter in the TLDG group, there were no significant differences in complication rates^27^. F H Ma *et al.* showed that regarding blood loss, surgical complications, post-op stay, harvested lymph nodes, LADG and TLDG have no difference^13^. These results indicate a lack of consensus regarding the superiority of TLDG in terms of complications, suggesting that further research is needed to reach a consensus.

In our study, we investigated the short-term complications and resected lymph nodes after both LADG and TLDG. Our center has rich experience in laparoscopic gastrectomy with a high volume of over 1,000 laparoscopic gastrectomy cases per year in the past 5 years. We collected laparoscopic distal gastrectomy cases from 2017 to 2021, which avoids the bias of including early laparoscopic cases with less experience. This study is one of the latest studies that present the short-term and long-term outcomes of LADG and TLDG, which might indicate the latest results and could be different from the early previous studies. Notably, contrary to some prior reports, our analysis leans toward favoring TLDG in certain complication comparisons. In our overall comparison of complications, we observed a significantly lower complication rate in TLDG compared to LADG. We also found that wound infection rate is higher in LADG than TLDG, which is within our expectation, for the longer incision in LADG is more like open surgery with a higher infection risk. For anastomosis-related complications, TLDG also exerts significant better outcomes than LADG. Although for grade III-V complications there is no significant difference, postoperative stay also indicates that TLDG is better in shorter postoperative time, which might be also related to lower complication rates. We found that all TLDG anastomosis methods could demonstrate better outcomes on postoperative hospital stay. These results above indicate that with sufficient experience, comparatively less invasiveness may lead to better recovery and prove TLDG might be a better option regarding overall and anastomosis-related complications.

Since Billroth-I, Billroth-II, Roux-en-Y and Uncut were applied in laparoscopic distal gastrectomy, no agreement on advantages and disadvantages of 4 anastomosws were reached regarding complications and postoperative stay. And now there are no publications comparing 4 anastomoses with each other in a LADG or TLDG way until the present one. In this study, we also compared different anastomoses with each other in LADG subgroup, TLDG subgroup and all groups. No difference was observed in the comparison of complications. In the postoperative stay, after combining the two subgroups, we noticed that the postoperative hospital stay of RY was significantly longer than that of B-II and Uncut. However, postoperative stay is a comparatively weaker parameter compared to complications which could be influenced by other factors, so this might not strongly suggest superiority of B-II or Uncut. Generally, these results showed that different anastomosis methods with experienced surgeons will not affect overall complication rates, grade III-V complication rates, anastomosis-related complication rates and postoperative hospital stay. The evidence was not quite convincing and consistent.

We also conducted multivariate analysis of variance to detect if different stages may affect the complications rates. Results turned out that different T or N stages had no influence on overall complication rates, anastomosis-related complication rate and grade III-V complication rate, while significant differences were usually observed between TLDG and LADG in most analysis, regardless of other factors. This piece of evidence combined with previous analysis suggested that TLDG might be safe and feasible not only for early gastric cancer but also for advanced gastric cancer.

Operation time is also one of the factors that indicates the quality of surgery, but it is comparatively weaker. Through our study we could notice LADG cases have longer operation time than TLDG cases, which is within our expectations. With a wider incision for extracorporeal gastrointestinal anastomosis, LADG cases may need more time to complete the procedure. Moreover, it is a retrospective study and most LADG cases were completed in the early time during the period, so this also might influence the operation time. But generally, TLDG has shorter operation time than LADG cases.

There is no significant statistical difference in the 5-year OS between the TLDG group and the LADG group, whether considering the OS of all patients or conducting OS analysis stratified by staging. However, overall, the OS of the TLDG group showed a slightly better trend than that of the LADG group. Nevertheless, this small difference may be attributed to the fact that experienced doctors in our center tend to prefer TLDG over LADG. But prognosis of both LADG and TLDG groups was acceptable and consistent with other reports.

We understand there are some limitations in our study. This is a retrospective study enrolled in a single gastric cancer center. We included a single center in the study but this may not be sufficient to indicate the difference. Also, even though we included 4 anastomoses with LADG and TLDG in the study, the number of some certain cases is not enough to provide solid evidence because of the preference of different methods. What's more, different equipment, habits, standards and policies of different surgeons may also result in bias. Therefore, a large-scale RCT may be the key to solving the problem. KLASS-07(CKLASS-01) is a large-scale RCT to compare perioperative and clinicopathologic outcomes between LADG and TLDG which is now ongoing. Recent KLASS-07 studies have shown that no significant differences in postoperative results between LADG and TLDG, but final results are still awaiting^10^, ^12^.

## Conclusion

Our study indicates that TLDG is in favor of overall complication rate, anastomosis-related complication rate, opostoperative hospital stay and operation time than LADG. No significant difference was observed in Grade III-V complications, resected lymph nodes and prognosis between TLDG and LADG. Billroth-I, Billroth-II, Roux-en-Y and Uncut Roux-en-Y did not demonstrate convincing evidence of being superior in complications rates, postoperative stay and harvested lymph nodes than each other.

## Supplementary Material

Supplementary tables.

## Figures and Tables

**Table 1:** The detailed information of patients enrolled in this study from NMUH.

Variable	Total	LADG	TLDG	*p*-Value
Age	59.50±10.58	58.34±10.95	59.69±10.51	0.872
Gender				0.543
Male	786	111	675	
Female	435	56	379	
Anastomosis				<0.001^*^
B-Ⅰ	12	1	11	
B-Ⅱ	753	49	704	
R-Y	203	102	101	
Uncut	253	15	238	
T Stage				0.029^*^
T1	626	85	541	
T2	163	24	139	
T3	277	33	244	
T4	155	40	115	
N Stage				0.807
N0	685	96	589	
N1	175	22	153	
N2	152	22	130	
N3a	142	20	122	
N3b	67	8	59	
Tumor Size				0.995
≤2.5cm	687	94	593	
>2.5cm	534	73	461	
Harvested lymph nodes	42.08±10.75	40.72±10.81	42.29±10.74	0.7970

**Table 2A:** The overall complication rates of enrolled cases from NMUH.

	Anastomosis	Cases	Complication cases	Complication rate
LADG	B-I	1	1	100%
	B-II	49	8	16.33%
	RY	102	18	17.65%
	uncut	15	2	13.33%
	Total	167	29	17.37%
TLDG	B-I	11	1	9.09%
	B-II	704	85	12.07%
	RY	101	11	10.89%
	uncut	238	16	6.72%
	Total	1054	113	10.72%
LADG+TLDG	B-I	12	2	16.67%
	B-II	753	93	12.35%
	RY	203	29	14.29%
	uncut	253	18	7.11%
	Total	1221	142	11.63%

**Table 2B:** Analysis of overall complications between LADG and TLDG (*p<0.05).

	LADG	Rate	TLDG	Rate	*p*-Value
LADG vs TLDG	B-I	100%	B-I	9.09%	/
	B-II	16.33%	B-II	12.07%	0.382
	RY	17.65%	RY	10.89%	0.169
	Uncut	13.33%	Uncut	6.72%	0.654
	Total	17.37%	Total	10.72%	0.013^*^

**Table 3A:** The anastomosis-related complication rates of all cases from NMUH. Analysis of anastomosis-related complications among different anastomoses.

	Anastomosis	Cases	Complication cases	Complication rate
LADG	B-I	1	1	100%
	B-II	49	2	4.08%
	RY	102	4	3.92%
	Uncut	15	1	6.67%
	Total	167	8	4.79%
TLDG	B-I	11	0	0%
	B-II	704	8	1.13%
	RY	101	3	2.97%
	Uncut	238	1	0.42%
	Total	1054	12	1.13%
LADG+TLDG	B-I	12	1	8.33%
	B-II	753	10	1.33%
	RY	203	7	3.45%
	Uncut	253	2	0.79%
	Total	1221	20	1.64%

**Table 3B:** The anastomosis-related complication rates of all cases from NMUH. Analysis of anastomosis-related complications in LADG between TLDG (*p<0.05).

	LADG	Rate	TLDG	Rate	*p*-Value
LADG vs TLDG	B-I	100%	B-I	0%	/
	B-II	4.08%	B-II	1.13%	0.134
	RY	3.92%	RY	2.97%	1.000
	Uncut	6.67%	Uncut	0.42%	0.115
	Total	4.79%	Total	1.13%	0.002^*^

**Table 4A:** The grade III-V complication rates of all enrolled cases from NMUH. The overall information of grade III-V complication rates of all enrolled cases from NMUH.

	Anastomosis	Cases	Complication (III-V) cases	Complication (III-V) rate
LADG	B-I	1	1	100%
	B-II	49	1	2.04%
	RY	102	2	1.96%
	Uncut	15	1	6.67%
	Total	167	5	2.99%
TLDG	B-I	11	1	9.09%
	B-II	704	21	2.98%
	RY	101	4	3.96%
	Uncut	238	5	2.10%
	Total	1054	31	2.94%
LADG+TLDG	B-I	12	2	16.67%
	B-II	753	22	2.92%
	RY	203	6	2.96%
	Uncut	253	6	2.37%
	Total	1221	36	2.95%

**Table 4B:** The grade III-V complication rates of all enrolled cases from NMUH. Analysis of grade III-V complications in LADG and TLDG (*p<0.05).

	LADG	Rate	TLDG	Rate	*p*-Value
LADG vs TLDG	B-I	100%	B-I	9.09%	/
	B-II	2.04%	B-II	2.98%	1.000
	RY	1.96%	RY	3.96%	0.670
	Uncut	6.67%	Uncut	2.10%	0.310
	Total	2.99%	Total	2.94%	1.000

**Table 5A:** The postoperative hospital stay of enrolled cases from NMUH. The overall information of postoperative hospital stay of enrolled cases from NMUH.

	Anastomosis	Cases	Post-op stay (d)
LADG	B-I	1	28
	B-II	49	8(7,10)
	RY	102	10(8.25,11)
	Uncut	15	8(8,9)
	Total	167	9(8,11)
TLDG	B-I	11	9(8,10)
	B-II	704	7(7,9)
	RY	101	8(7,9)
	Uncut	238	7(7,8)
	Total	1054	8(7,9)
LADG+TLDG	B-I	12	9.5(8,10.25)
	B-II	753	8(7,9)
	RY	203	9(8,10)
	Uncut	253	8(7,9)
	Total	1221	8(7,9)

**Table 5B:** The postoperative hospital stay of enrolled cases from NMUH. Analysis of postoperative stay between LADG and TLDG (*p<0.05).

	LADG	Post-op stay(d)	TLDG	Post-op stay(d)	*p*-Value
LADG vs TLDG	B-I	28	B-I	9(8,10)	/
	B-II	8(7,10)	B-II	7(7,9)	0.0260^*^
	RY	10(8.25,11)	RY	8(7,9)	<0.0001^*^
	Uncut	8(8,9)	Uncut	7(7,8)	0.0059^*^
	Total	9(8,11)	Total	8(7,9)	<0.0001^*^

**Table 6A:** The operation time of enrolled cases from NMUH. The overall information of operation time of enrolled cases from NMUH.

	Anastomosis	Cases	Operation time(min)
LADG	B-I	1	191
	B-II	49	187.31±50.91
	RY	102	238.6±59.55
	Uncut	15	199.07±43.72
	Total	167	219.71±60.34
TLDG	B-I	11	178.73±40.55
	B-II	704	173.57±36.74
	RY	101	178.32±47.79
	Uncut	238	173.79±35.99
	Total	1054	174.13±37.78
LADG+TLDG	B-I	12	179.75±38.82
	B-II	753	174.46±37.93
	RY	203	208.61±61.78
	Uncut	253	174.08±36.07
	Total	1221	180.36±44.42

**Table 6B:** The operation time of enrolled cases from NMUH. Analysis of operation time between LADG and TLDG (*p<0.05).

	LADG	Operation time(min)	TLDG	Operation time(min)	*p*-Value
LADG vs TLDG	B-I	191	B-I	178.73±40.55	/
	B-II	187.31±50.91	B-II	173.57±36.74	0.0693
	RY	238.6±59.55	RY	178.32±47.79	<0.0001
	Uncut	199.07±43.72	Uncut	173.79±35.99	0.0443
	Total	219.71±60.34	Total	174.13±37.78	<0.0001
