# Supplementary material for: Comparison of Short-term and Long-term Outcomes after Different Reconstructions between Totally Laparoscopic Distal Gastrectomy and Laparoscopic-assisted Distal Gastrectomy for Gastric Cancer: a Retrospective Analysis at a High-volume Center
Source: J Cancer. 2024 Jul 16;15(15):4893–901. doi: 10.7150/jca.97786 (PMC11310888; doi:10.7150/jca.97786)
Supplement: Supplementary file 1 — Supplementary tables. [file jcav15p4893s1.pdf]

Supplementary materials

Table S1: The comparisons of overall complications, anastomosis-related complications, grade III-V complications of multiple groups in LADG, TLDG and the combined data (*p<0.05).

	Anastomosis	Overall complication rates	p-Value	Anastomosis-related complications rates	p-Value	Grade III-V complication rates	p-Value
LADG	B-II	16.33%	0.91	4.08%	0.884	2.04%	0.529
	RY	17.65%		3.92%		1.96%	
	uncut	13.33%		6.67%		6.67%	
TLDG	B-I	9.09%	0.148	0%	0.131	9.09%	0.49
	B-II	12.07%		1.13%		2.98%	
	RY	10.89%		2.97%		3.96%	
	uncut	6.72%		0.42%		2.10%	
LADG+TLDG	B-II	12.35%	0.066	1.33%	0.052	2.92%	0.892
	RY	14.29%		3.45%		2.96%	
	uncut	7.11%		0.79%		2.37%	

Table S2: Analysis of different kinds of complications in LADG and TLDG (*p<0.05).

Complication	LADG	Rate	TLDG	Rate	p-Value
Duodenal stump leakage	3/167	1.80%	4/1054	0.38%	0.058
Anastomotic leakage	3/167	1.80%	3/1054	0.28%	0.037*
Anastomotic bleeding	2/167	1.20%	5/1054	0.47%	0.246
Intraabdominal	2/167	1.20%	10/1054	0.95%	0.768

bleeding					
Intestinal obstruction	0/167	0%	3/1054	0.28%	1.000
Abdominal infection	4/167	0.60%	12/1054	1.13%	0.225
Seroperitoneum	1/167	0.60%	15/1054	1.42%	0.338
Wound infection	4/167	2.40%	4/1054	0.38%	0.013*
Lymphatic fistula	1/167	0.60%	6/1054	0.57%	1.000
Anemia	1/167	0.60%	3/1054	0.28%	0.445
Pulmonary	4/167	0.60%	22/1054	0.85%	0.801
Mobility Disorder	2/167	1.20%	7/1054	0.66%	0.793
Cardiac	1/167	0.60%	0/1054	0%	0.137
Hepatobiliary	2/167	1.20%	13/1054	1.23%	1.000
Other gastrointestinal	3/167	1.80%	10/1054	0.95%	0.558
Urinary and renal	0/167	0%	5/1054	0.85%	1.000
Thrombosis	0/167	0%	3/1054	0.47%	1.000

Table S3: Univariate and multivariate analysis of variance in overall complications (*p<0.05).

Variables	Category	Overall Complication		Univariate analysis	Multivariate analysis
		Yes, n	No, n	p-Value	p-Value
TLDG or LADG	LADG	29	138	0.013*	<0.001*
	TLDG	113	941		
T stage	T1	65	561	0.551	/
	T2	15	148		0.334
	T3	35	242		0.931

	T4	19	136		0.743
N stage	N0	74	611	0.571	/
	N1	18	157		0.981
	N2	23	129		0.175
	N3a	18	124		0.649
	N3b	9	58		0.605

Table S4: Univariate and multivariate analysis of variance in anastomosis-related complications (**p*<0.05).

Variables	Category	Overall Complication		Univariate analysis	Multivariate analysis
		Yes, n	No, n	*p*-Value	*p*-Value
TLDG or LADG	LADG	12	1042	0.002*	0.002*
	TLDG	8	159		
T stage	T1	7	619	0.586	/
	T2	2	161		0.837
	T3	5	272		0.643
	T4	4	151		0.260
N stage	N0	10	675	0.555	/
	N1	2	173		0.695
	N2	5	147		0.359
	N3a	2	140		0.632
	N3b	1	66		0.750

Table S5: Univariate and multivariate analysis of variance in grade III-V

complications (*p<0.05).

Variables	Category	Grade III-V complications		Univariate analysis	Multivariate analysis
		Yes, n	No, n	p	p
TLDG or LADG	TLDG	31	1023	1.000	0.993
	LADG	5	162		
T stage	T1	17	609	0.783	/
	T2	3	160		0.618
	T3	9	268		0.312
	T4	5	150		0.202
N stage	N0	23	662	0.821	/
	N1	4	171		0.356
	N2	5	147		0.618
	N3a	3	139		0.198
	N3b	1	66		0.225

Table S6: The comparisons of multiple groups of postoperative stay in LADG, TLDG and the combined data (*p<0.05).

	Anastomosis	Post-op stay(d)	p-Value
LADG	B-II	8(7,10)	<0.001*
	RY	10(8.25,11)	
	Uncut	8(8,9)	
TLDG	B-I	9(8,10)	<0.001*
	B-II	7(7,9)	
	RY	8(7,9)	
	Uncut	7(7,8)	

LADG+TLDG	B-I	9.5(8,10.25)	<0.001*
	B-II	8(7,9)	
	RY	9(8,10)	
	Uncut	8(7,9)	

Table S7: The two components analysis of postoperative stay among different anastomosis in LADG (*p<0.016).

	Anastomosis	Post-op stay(d)	Anastomosis	Post-op stay(d)	*p*-Value
LADG	B-II	8(7,10)	RY	10(8.25,11)	<0.001*
	B-II	8(7,10)	Uncut	8(8,9)	0.3670
	RY	10(8.25,11)	Uncut	8(8,9)	0.0323

Table S8: The two components analysis of postoperative stay among different anastomosis in TLDG (*p<0.0125).

	Anastomosis	Post-op stay(d)	Anastomosis	Post-op stay(d)	*p*-Value
TLDG	B-I	9(8,10)	B-II	7(7,9)	0.0045*
	B-I	9(8,10)	RY	8(7,9)	0.0284
	B-I	9(8,10)	Uncut	7(7,8)	0.0027*
	B-II	7(7,9)	RY	8(7,9)	0.2170
	B-II	7(7,9)	Uncut	7(7,8)	0.8056
	RY	8(7,9)	Uncut	7(7,8)	0.1822

Table S9: The two components analysis of postoperative stay among different

anastomosis in combination of LADG and TLDG (*p<0.016).

	Anastomosis	Post-op stay(d)	Anastomosis	Post-op stay(d)	p-Value
LADG+TLDG	B-II	8(7,9)	RY	9(8,10)	<0.0001*
	B-II	8(7,9)	Uncut	8(7,9)	0.9997
	RY	9(8,10)	Uncut	8(7,9)	<0.0001*

Table S10: The comparisons of multiple groups of operation time in LADG and TLDG (*p<0.05).

	Anastomosis	Operation time(min)	p-Value
LADG	B-II	187.31±50.91	<0.0001
	RY	238.6±59.55	
	Uncut	199.07±43.72	
TLDG	B-I	178.73±40.55	0.6651
	B-II	173.57±36.74	
	RY	178.32±47.79	
	Uncut	173.79±35.99	

Table S11: The two components analysis of operation time among different anastomosis in LADG (*p<0.0125).

	Anastomosis	Operation time(min)	Anastomosis	Operation time(min)	p-Value
LADG	B-II	187.31±50.91	RY	238.6±59.55	<0.0001
	B-II	187.31±50.91	Uncut	199.07±43.72	0.3890

	RY	238.6±59.55	Uncut	199.07±43.72	0.0051
